# Supplementary material for: The Elevational Gradient of Bird Beta Diversity in the Meili Snow Mountains, Yunnan Province, China
Source: Animals (Basel). 2023 May 8;13(9):1567. doi: 10.3390/ani13091567 (PMC10177178; doi:10.3390/ani13091567)
Supplement: Supplementary file 1 [file animals-13-01567-s001.zip › animals-2280323-supplementary.pdf]

SUPPORTING INFORMATION

Table S1 Elevation bands and species distribution of birds on Meili Snow Mountain.

Species scientific name	Common name	2027-2249(m)	2250-2499(m)	2500-2749(m)	2750-2999(m)	3000-3249(m)	3250-3499(m)	3500-3749(m)	3750-3944(m)
Phylloscopus trochiloides	Greenish Warbler	0	2	4	7	12	13	9	2
Carpodacus nipalensis	Dark-breasted Rosefinch	0	1	0	0	0	0	0	0
Mycerobas carnipes	White-winged Grosbeak	0	0	0	0	0	0	6	0
Phylloscopus davisoni	Kloss's Leaf Warbler	0	0	0	0	0	0	2	0
Mycerobas melanozanthos	Spot-winged Grosbeak	0	0	6	0	8	0	0	0
Garrulax bieti	White-speckled Laughingthrush	0	0	0	0	0	0	2	0
Chaimarrornis leucocephalus	White-capped Water Redstart	1	0	1	0	1	0	2	0
Hodgsonius phoenicuroides	White-bellied Redstart	0	0	1	0	0	0	0	0
Chrysolophus amherstiae	Lady Amherst's Pheasant	0	9	17	13	3	3	0	1
Rhipidura albicollis	White-throated Fantail	0	0	1	0	0	0	0	0
Motacilla alba	White Wagtail	1	0	1	0	6	0	4	0
Yuhina diademata	White-collared Yuhina	0	0	0	4	131	15	6	0
Alcippe vinipectus	White-browed Fulvetta	0	0	6	10	4	46	11	8
Carpodacus thura	Chinese White-browed Rosefinch	0	0	0	0	0	0	0	2
Sitta himalayensis	White-tailed Nuthatch	0	0	2	0	0	0	0	0
Picumnus innominatus	Speckled Piculet	0	0	2	1	0	0	0	0
Pomatorhinus erythrocnemis	Black-streaked Scimitar Babbler	2	10	7	2	4	0	0	0
Turdus mupinensis	Chinese Thrush	0	0	0	0	1	0	0	0

Moupinia poecilotis	Rufous-tailed Babbler	0	1	0	0	0	0	0	0
Phoenicurus auroreus	Daurian Redstart	6	11	2	16	22	11	0	0
Phylloscopus pulcher	Buff-barred Warbler	0	0	4	1	23	27	29	35
Garrulax elliotii	Elliot's Laughingthrush	0	0	6	0	4	62	31	6
Ficedula strophiata	Rufous-gorgeted Flycatcher	0	0	0	0	3	2	4	0
Cuculus canorus	Common Cuckoo	0	0	1	0	0	0	0	0
Parus major	Cinereous Tit	3	29	5	12	0	0	0	0
Cuculus sparverioides	Large Hawk Cuckoo	0	0	0	0	5	0	0	0
Garrulax maximus	Giant Laughingthrush	0	0	0	4	5	18	15	2
Psittacula derbiana	Lord Derby’s Parakeet	0	2	0	2	2	7	5	4
Corvus macrorhynchos	Large-billed Crow	3	2	0	2	4	0	7	0
Upupa epops	Common Hoopoe	0	0	0	0	1	0	0	0
Pteruthius xanthochlorus	Green Shrike Babbler	0	0	2	0	0	0	0	0
Pericrocotus brevirostris	Short-billed Minivet	0	11	64	12	22	4	0	0
Culicicapa ceylonensis	Grey-headed Canary Flycatcher	1	0	3	0	0	0	0	0
Accipiter trivirgatus	Crested Goshawk	0	0	0	1	0	0	0	0
Certhia himalayana	Bar-tailed Treecreeper	0	0	0	1	2	0	1	2
Phylloscopus reguloides	Claudia's Leaf Warbler	4	22	31	26	47	34	35	17
Parus dichrous	Grey-crested Tit	0	2	0	0	15	18	10	4
Cinclus pallasii	Brown Dipper	2	0	1	0	2	0	0	0
Pyrrhula nipalensis	Brown Bullfinch	0	0	0	0	0	8	0	0
Phylloscopus fuscatus	Dusky Warbler	3	12	4	0	8	1	6	1
Parus montanus	Willow Tit	0	7	0	0	0	0	0	0
Garrulax affinis	Black-faced Laughingthrush	0	0	0	0	0	5	2	0
Hypsipetes leucocephalus	Black Bulbul	2	21	35	2	8	0	2	0
Parus rubidiventris	Rufous-vented Tit	0	0	0	1	12	11	19	18
Delichon nipalensis	Nepal House Martin	0	0	60	0	100	0	0	0
Saxicola torquata	Siberian Stonechat	0	0	0	0	1	0	0	0
Dicrurus macrocercus	Black Drongo	0	1	2	0	0	0	0	0

Aegithalos bonvaloti	Black-browed Bushtit	0	55	36	9	17	19	0	0
Carduelis ambigua	Black-headed Greenfinch	0	32	40	0	0	0	0	0
Pteruthius flaviscapis	Blyth's Shrike Babbler	0	0	0	1	0	0	0	0
Luscinia calliope	Siberian Rubythroat	0	0	0	0	1	0	0	0
Ficedula parva	Taiga Flycatcher	8	0	2	2	1	0	0	0
Carpodacus pulcherrimus	Himalayan Beautiful Rosefinch	0	0	1	0	25	4	0	0
Rhyacornis fuliginosus	Plumbeous Water Redstart	18	1	4	0	5	0	0	0
Tarsiger cyanurus	Orange-flanked Bluetail	0	0	0	0	0	6	7	4
Dicaeum ignipectus	Fire-breasted Flowerpecker	2	21	4	3	3	0	0	0
Pyrrhocorax pyrrhocorax	Red-billed Chough	2	0	0	0	2	0	0	0
Conostoma aemodium	Great Parrotbill	0	0	0	0	0	0	0	8
Phylloscopus affinis	Tickell's Leaf Warbler	0	0	0	0	0	0	2	1
Rhipidura hypoxantha	Citrine Canary-Flycatcher	0	0	0	2	0	1	4	0
Dicaeum melanozanthum	Yellow-bellied Flowerpecker	0	0	1	1	2	0	0	0
Emberiza elegans	Yellow-throated Bunting	0	5	2	2	1	0	0	0
Mycerobas affinis	Collared Grosbeak	0	0	0	0	0	0	0	5
Dendrocopos darjellensis	Darjeeling Woodpecker	0	0	0	0	0	2	0	0
Sylviparus modestus	Yellow-browed Tit	0	0	2	11	0	0	0	0
Phylloscopus inornatus	Yellow-browed Warbler	0	9	6	10	5	16	1	4
Pycnonotus xanthorrhous	Brown-breasted Bulbul	45	113	43	0	0	0	0	0
Phylloscopus proregulus	Pallas's Leaf Warbler	1	0	7	10	17	38	6	5
Lanius tephronotus	Grey-backed Shrike	0	3	5	1	8	0	0	0
Garrulax cineraceus	Moustached Laughingthrush	0	2	5	0	0	0	0	0
Zosterops palpebrosa	Oriental White-eye	0	7	14	0	0	0	0	0
Pericrocotus solaris	Grey-chinned Minivet	0	4	0	0	0	0	0	0
Motacilla cinerea	Grey Wagtail	1	0	3	1	3	0	1	0
Ficedula tricolor	Slaty-blue Flycatcher	0	0	1	0	0	0	1	0
Saxicola ferrea	Grey Bushchat	0	0	3	0	0	0	0	0
Emberiza cia	Godlewski's Bunting	7	18	5	2	6	2	0	0

Turdus rubrocanus	Chestnut Thrush	0	0	0	0	1	1	3	3
Pyrrhula erythaca	Grey-headed Bullfinch	0	0	2	13	4	3	26	9
Picus canus	Grey-headed Woodpecker	0	0	0	2	2	1	0	0
Liocichla phoenicea	Red-faced Liocichla	0	1	0	0	0	0	0	0
Cephalopyrus flammiceps	Fire-capped Tit	0	0	0	5	0	0	0	0
Certhia hodgsoni	Hodgson's Treecreeper	0	0	0	0	4	4	9	2
Phylloscopus borealis	Arctic Warbler	0	0	0	2	0	0	0	0
Troglodytes troglodytes	Eurasian Wren	0	0	0	0	0	0	4	2
Seicercus burkii	Green-crowned Warbler	0	1	4	7	7	2	5	1
Tarsiger chrysaeus	Golden Bush Robin	0	0	0	0	0	1	0	0
Phoenicurus frontalis	Blue-fronted Redstart	0	2	0	0	0	0	10	0
Aethopyga gouldiae	Mrs Gould's Sunbird	8	8	19	12	10	18	10	2
Monticola solitarius	Blue Rock Thrush	2	0	0	0	0	0	0	0
Luscinia brunnea	Indian Blue Robin	0	0	0	1	1	0	0	0
Sitta nagaensis	Chestnut-vented Nuthatch	0	1	10	11	26	15	9	0
Parus monticolus	Green-backed Tit	0	4	14	10	15	0	3	0
Aethopyga nipalensis	Green-tailed Sunbird	0	0	0	0	0	0	1	0
Passer montanus	Eurasian Tree Sparrow	0	58	0	0	0	0	0	0
Parus ater	Coal Tit	0	0	0	0	4	75	2	0
Carpodacus erythrinus	Common Rosefinch	0	8	11	0	40	0	0	0
Streptopelia orientalis	Oriental Turtle Dove	6	39	21	9	20	7	4	0
Niltava banyumas	Hill Blue Flycatcher	3	0	2	2	0	0	0	0
Anthus sylvanus	Upland Pipit	0	1	0	0	0	0	0	0
Passer rutilans	Russet Sparrow	32	41	0	0	0	0	0	0
Pucrasia macrolopha	Koklass Pheasant	0	0	0	0	0	1	0	0
Anthus hodgsoni	Olive-backed Pipit	0	0	0	0	23	0	0	0
Accipiter virgatus	Besra	0	0	0	0	0	1	0	0
Garrulus glandarius	Eurasian Jay	0	0	0	1	0	0	0	0
Muscicapa thalassina	Verditer Flycatcher	0	1	0	0	1	0	0	0
Turdus merula	Chinese Blackbird	0	0	0	2	0	0	0	0

Phylloscopus magnirostris	Large-billed Leaf Warbler	0	0	1	0	1	4	0	0
Pica pica	Common Magpie	2	5	0	0	0	0	0	0
Ficedula westermanni	Little Pied Flycatcher	0	1	0	0	0	0	0	0
Corvus corone	Carrion Crow	3	0	2	0	3	2	0	0
Treron sphenura	Wedge-tailed Green Pigeon	5	2	2	0	2	0	0	0
Nucifraga caryocatactes	Spotted Nutcracker	0	6	36	18	11	5	1	1
Ficedula hodgsonii	Slaty-backed Flycatcher	0	0	1	2	4	2	0	0
Columba leuconota	Snow Pigeon	0	2	0	2	129	0	18	0
Ithaginis cruentus	Blood Pheasant	0	0	0	0	0	0	2	0
Columba rupestris	Hill Pigeon	48	4	11	0	0	0	0	0
Ptyonoprogne rupestris	Eurasian Crag Martin	9	28	0	0	0	0	0	0
Ficedula sapphira	Sapphire Flycatcher	0	0	0	1	0	0	0	0
Zoothera dixoni	Long-tailed Thrush	0	0	0	0	0	1	0	0
Uragus sibiricus	Long-tailed Rosefinch	0	4	1	3	0	0	0	0
Pericrocotus ethologus	Long-tailed Minivet	0	0	0	0	2	2	6	0
Parus palustris	Marsh Tit	0	3	2	6	12	15	0	0
Myiophoneus caeruleus	Blue Whistling Thrush	6	3	3	3	2	0	0	0
Phylloscopus subaffinis	Buff-throated Warbler	0	2	0	0	0	0	2	0
Niltava sundara	Rufous-bellied Niltava	0	0	0	1	0	0	1	0
Dendrocopos hyperythrus	Rufous-bellied Woodpecker	0	0	0	0	1	1	2	3
Pomatorhinus ruficollis	Streak-breasted Scimitar Babbler	0	4	8	4	0	0	0	0
Phylloscopus armandii	Yellow-streaked Warbler	0	0	2	0	2	0	0	0
Alcippe ruficapilla	Spectacled Fulvetta	0	3	0	0	0	0	0	0
Yuhina occipitalis	Rufous-vented Yuhina	0	0	0	0	10	0	41	4
Ficedula hyperythra	Snowy-browed Flycatcher	0	0	0	0	4	0	1	0
Prunella strophiata	Rufous-breasted Accentor	0	0	0	0	0	0	11	0

Table S2 Functional traits of each bird species on Meili Snow Mountain.

Scientific name	Common name	Diet-Inv	Diet-Vend	Diet-Vect	Diet-Vfish	Diet-Vunk	Diet-Scav	Diet-Fruit	Diet-Nect	Diet-Seed	Diet-PlantO	ForStrat-watbelowsurf	ForStrat-wataroundsurf	ForStrat-ground	ForStrat-understory	ForStrat-midhigh	ForStrat-canopy	ForStrat-aerial	BodyMass-Value
Ithaginis cruentus	Blood Pheasant	20	0	0	0	0	0	20	0	10	50	0	0	80	20	0	0	0	530.67
Pucrasia macrolopha	Koklass Pheasant	20	0	0	0	0	0	30	0	30	20	0	0	100	0	0	0	0	1050.47
Chrysolophus amherstiae	Lady Amherst's Pheasant	40	0	0	0	0	0	10	0	0	50	0	0	100	0	0	0	0	732.47
Picumnus innominatus	Speckled Piculet	100	0	0	0	0	0	0	0	0	0	0	0	0	50	50	0	0	10.2
Dendrocopos hyperythrus	Rufous-bellied Woodpecker	70	0	0	0	0	0	0	20	0	10	0	0	0	0	50	50	0	46.49
Dendrocopos darjellensis	Darjeeling Woodpecker	100	0	0	0	0	0	0	0	0	0	0	0	0	33	33	33	0	70.66
Picus canus	Grey-headed Woodpecker	70	0	0	0	0	0	10	10	10	0	0	0	100	0	0	0	0	137
Upupa epops	Common Hoopoe	80	0	20	0	0	0	0	0	0	0	0	0	100	0	0	0	0	66.93
Cuculus sparverioides	Large Hawk Cuckoo	90	0	0	0	0	0	10	0	0	0	0	0	0	0	40	60	0	151
Cuculus canorus	Common Cuckoo	90	0	0	0	0	0	10	0	0	0	0	0	20	20	40	20	0	111.36
Psittacula derbiana	Lord Derby’s Parakeet	0	0	0	0	0	0	50	0	50	0	0	0	0	33	33	33	0	251
Columba rupestris	Hill Pigeon	10	0	0	0	0	0	0	0	70	20	0	0	80	20	0	0	0	245.41
Columba leuconota	Snow Pigeon	0	0	0	0	0	0	0	0	70	30	0	0	80	20	0	0	0	284
Streptopelia orientalis	Oriental Turtle Dove	0	0	0	0	0	0	20	0	40	40	0	0	100	0	0	0	0	232.94
Treron sphenurus	Rusty Tinamou	0	0	0	0	0	0	100	0	0	0	0	0	20	0	40	40	0	210
Accipiter trivirgatus	Crested Goshawk	20	60	20	0	0	0	0	0	0	0	0	0	33	33	33	0	0	265.04
Accipiter virgatus	Besra	20	60	20	0	0	0	0	0	0	0	0	0	50	0	30	20	0	116.99
Lanius tephronotus	Grey-backed Shrike	80	10	10	0	0	0	0	0	0	0	0	0	100	0	0	0	0	46.35
Garrulus glandarius	Eurasian Jay	40	10	0	0	0	0	30	0	20	0	0	0	60	20	20	0	0	159.46

Pica pica	Common Magpie	20	20	20	0	0	20	10	0	10	0	0	0	70	10	10	0	10	217.48
Nucifraga caryocatactes	Spotted Nutcracker	30	10	0	0	0	0	0	0	60	0	0	0	40	20	20	20	0	182.51
Pyrrhocorax pyrrhocorax	Red-billed Chough	50	0	0	0	10	10	10	0	20	0	0	0	70	0	30	0	0	276.68
Corvus corone	Carrion Crow	30	20	20	0	0	20	0	0	10	0	0	0	90	0	0	0	10	570
Corvus macrorhynchos	Large-billed Crow	20	10	10	0	0	30	20	0	10	0	0	0	100	0	0	0	0	513.14
Pericrocotus solaris	Grey-chinned Minivet	100	0	0	0	0	0	0	0	0	0	0	0	0	0	0	100	0	14.5
Pericrocotus ethologus	Long-tailed Minivet	80	0	0	0	0	0	10	0	0	10	0	0	0	0	0	80	20	18.97
Pericrocotus brevirostris	Short-billed Minivet	80	0	0	0	0	0	0	0	0	20	0	0	0	33	33	33	0	16.49
Chelidorhynx hypoxantha	Slaty-breasted Tinamou	100	0	0	0	0	0	0	0	0	0	0	0	10	40	40	10	0	14.7
Rhipidura albicollis	White-throated Fantail	100	0	0	0	0	0	0	0	0	0	0	0	0	40	40	20	0	12.9
Dicrurus macrocercus	Black Drongo	40	10	10	0	0	10	0	20	10	0	0	0	0	0	33	33	33	48.3
Cinclus pallasii	Brown Dipper	100	0	0	0	0	0	0	0	0	0	40	40	20	0	0	0	0	76
Monticola solitarius	Blue Rock Thrush	40	10	20	0	0	0	20	0	10	0	0	0	80	0	20	0	0	53.65
Myophonus caeruleus	Brazilian Tinamou	60	0	10	0	0	0	30	0	0	0	0	0	100	0	0	0	0	157.98
Zoothera dixoni	Long-tailed Thrush	70	0	0	0	0	0	20	0	10	0	0	0	100	0	0	0	0	90
Turdus merula	Chinese Blackbird	50	0	0	0	10	0	20	0	20	0	0	0	60	20	20	0	0	102.73
Turdus rubrocanus	Chestnut Thrush	50	0	0	0	0	0	50	0	0	0	0	0	60	0	20	20	0	92.2
Turdus mupinensis	Chinese Thrush	40	0	0	0	0	0	50	0	10	0	0	0	40	20	20	20	0	62.54
Ficedula hodgsonii	Slaty-backed Flycatcher	50	0	0	0	0	0	50	0	0	0	0	0	0	50	50	0	0	10
Ficedula strophiata	Rufous-gorgeted Flycatcher	100	0	0	0	0	0	0	0	0	0	0	0	10	40	50	0	0	12.84
Ficedula hyperythra	Snowy-browed Flycatcher	90	0	0	0	0	0	10	0	0	0	0	0	0	80	20	0	0	8.2
Ficedula westermanni	Little Pied Flycatcher	100	0	0	0	0	0	0	0	0	0	0	0	0	0	40	60	0	7.8
Ficedula tricolor	Slaty-blue Flycatcher	100	0	0	0	0	0	0	0	0	0	0	0	33	33	33	0	0	8.26
Ficedula sapphira	Sapphire Flycatcher	100	0	0	0	0	0	0	0	0	0	0	0	0	50	50	0	0	7.8

Eumyias thalassinus	Tabon Megapode	70	0	0	0	0	0	30	0	0	0	0	0	20	0	40	40	0	18.1
Niltava sundara	Rufous-bellied Niltava	80	0	0	0	0	0	20	0	0	0	0	0	50	50	0	0	0	21.1
Cyornis banyumas	Taczanowski's Tinamou	100	0	0	0	0	0	0	0	0	0	0	0	0	50	50	0	0	14.5
Culicicapa ceylonensis	Grey-headed Canary Flycatcher	100	0	0	0	0	0	0	0	0	0	0	0	0	30	30	40	0	7.7
Luscinia calliope	Siberian Rubythroat	80	0	0	0	0	0	0	0	0	20	0	0	50	50	0	0	0	18.5
Luscinia brunnea	Indian Blue Robin	100	0	0	0	0	0	0	0	0	0	0	0	70	30	0	0	0	17.5
Tarsiger cyanurus	Orange-flanked Bluetail	80	0	0	0	0	0	10	0	10	0	0	0	50	50	0	0	0	13.43
Tarsiger chrysaeus	Golden Bush Robin	100	0	0	0	0	0	0	0	0	0	0	0	50	50	0	0	0	13.8
Phoenicurus auroreus	Daurian Redstart	60	0	0	0	0	0	0	0	30	10	0	0	50	50	0	0	0	16.2
Phoenicurus frontalis	Blue-fronted Redstart	100	0	0	0	0	0	0	0	0	0	0	0	0	33	33	33	0	15.77
Chaimarrornis leucocephalus	White-capped Water Redstart	80	0	0	0	0	0	10	0	10	0	0	0	80	20	0	0	0	30.04
Rhyacornis fuliginosa	Southern Cassowary	80	0	0	0	0	0	10	0	10	0	0	0	50	25	25	0	0	20.33
Hodgsonius phaenicuroides	Grey-headed Chachalaca	60	0	0	0	0	0	40	0	0	0	0	0	100	0	0	0	0	21.1
Saxicola torquatus	Sand Partridge	70	0	0	0	10	0	10	0	10	0	0	0	100	0	0	0	0	14.09
Saxicola ferreus	White-bellied Chachalaca	90	0	0	0	0	0	0	0	10	0	0	0	80	10	10	0	0	14.7
Sitta nagaensis	Chestnut-vented Nuthatch	70	0	0	0	0	0	0	0	30	0	0	0	60	20	20	0	0	14.7
Sitta himalayensis	White-tailed Nuthatch	70	0	0	0	0	0	0	0	30	0	0	0	0	20	50	30	0	14.28
Certhia himalayana	Bar-tailed Treecreeper	90	0	0	0	0	0	0	0	10	0	0	0	10	30	30	30	0	8.8
Troglodytes troglodytes	Eurasian Wren	60	0	10	10	0	0	10	0	10	0	0	0	50	50	0	0	0	9.74
Cephalopyrus flammiceps	Fire-capped Tit	60	0	0	0	0	0	0	20	0	20	0	0	0	20	40	40	0	7
Parus palustris	Marsh Tit	50	0	0	0	0	0	20	0	20	10	0	0	0	20	80	0	0	11.14
Parus montanus	Willow Tit	60	0	0	0	0	0	20	0	20	0	0	0	0	20	80	0	0	11.1
Parus rubidiventris	Rufous-vented Tit	80	0	0	0	0	0	0	0	20	0	0	0	0	0	40	60	0	11.83

Parus ater	Coal Tit	40	0	0	0	0	10	0	10	20	20	0	0	100	0	0	0	0	9.2
Parus dichrous	Grey-crested Tit	100	0	0	0	0	0	0	0	0	0	0	0	0	20	80	0	0	13.8
Parus major	Cinereous Tit	40	0	0	0	0	10	20	10	20	0	0	0	0	20	60	20	0	16.25
Parus monticolus	Green-backed Tit	50	0	0	0	0	0	20	0	20	10	0	0	0	30	30	40	0	13.95
Sylviparus modestus	Yellow-browed Tit	80	0	0	0	0	0	0	0	20	0	0	0	0	20	50	30	0	7.14
Aegithalos iouschistos	Solitary Tinamou	70	0	0	0	0	0	0	0	0	30	0	0	0	50	0	50	0	7
Hirundo rupestris	Chestnut-bellied Guan	100	0	0	0	0	0	0	0	0	0	0	0	40	40	20	0	0	20.4
Delichon nipalense	Chestnut-winged Chachalaca	100	0	0	0	0	0	0	0	0	0	0	0	0	0	0	20	80	14.96
Pycnonotus xanthorrhous	Brown-breasted Bulbul	20	0	0	0	0	0	60	0	20	0	0	0	50	50	0	0	0	26.9
Hypsipetes leucocephalus	Black Bulbul	20	0	0	0	0	0	40	20	0	20	0	0	0	10	20	70	0	51.8
Zosterops palpebrosus	Little Chachalaca	40	0	0	0	0	0	20	10	10	20	0	0	0	0	30	70	0	8.6
Phylloscopus fuscatus	Dusky Warbler	80	0	0	0	0	0	0	0	20	0	0	0	0	50	50	0	0	8.74
Phylloscopus affinis	Tickell's Leaf Warbler	100	0	0	0	0	0	0	0	0	0	0	0	50	50	0	0	0	6.96
Phylloscopus subaffinis	Buff-throated Warbler	100	0	0	0	0	0	0	0	0	0	0	0	30	10	30	30	0	6.2
Phylloscopus armandii	Yellow-streaked Warbler	100	0	0	0	0	0	0	0	0	0	0	0	33	33	33	0	0	9.4
Phylloscopus pulcher	Buff-barred Warbler	80	0	0	0	0	0	0	20	0	0	0	0	0	0	40	60	0	6.8
Phylloscopus proregulus	Pallas's Leaf Warbler	100	0	0	0	0	0	0	0	0	0	0	0	0	50	0	50	0	6
Phylloscopus inornatus	Yellow-browed Warbler	90	0	0	0	0	0	0	0	10	0	0	0	10	30	30	30	0	6.59
Phylloscopus borealis	Arctic Warbler	100	0	0	0	0	0	0	0	0	0	0	0	0	50	0	50	0	10.89
Phylloscopus trochiloides	Greenish Warbler	80	0	0	0	0	0	10	0	10	0	0	0	0	33	33	33	0	8.05
Phylloscopus magnirostris	Large-billed Leaf Warbler	100	0	0	0	0	0	0	0	0	0	0	0	0	0	20	80	0	11.6
Seicercus burkii	Green-crowned Warbler	100	0	0	0	0	0	0	0	0	0	0	0	0	0	100	0	0	7.3
Garrulax cineraceus	Moustached Laughingthrush	70	0	0	0	0	0	20	0	10	0	0	0	100	0	0	0	0	48.95

Garrulax bieti	White-speckled Laughingthrush	50	0	0	0	0	0	50	0	0	0	0	0	100	0	0	0	0	78.64
Garrulax maximus	Giant Laughingthrush	80	0	0	0	0	0	0	0	0	20	0	0	80	10	10	0	0	78.64
Garrulax elliotii	Elliot's Laughingthrush	80	0	0	0	0	0	0	0	0	20	0	0	50	50	0	0	0	45
Garrulax affinis	Black-faced Laughingthrush	30	0	0	0	0	0	70	0	0	0	0	0	40	40	20	0	0	72.66
Liocichla phoenicea	Red-faced Liocichla	40	0	0	0	0	0	30	0	30	0	0	0	50	50	0	0	0	49
Pomatorhinus erythrocnemis	Black-streaked Scimitar Babbler	100	0	0	0	0	0	0	0	0	0	0	0	100	0	0	0	0	58.15
Pomatorhinus ruficollis	Streak-breasted Scimitar Babbler	80	0	0	0	0	0	10	0	10	0	0	0	0	80	20	0	0	31.61
Chrysomma poecilotis	Baudo Guan	100	0	0	0	0	0	0	0	0	0	0	0	0	50	50	0	0	18.71
Pteruthius flaviscapis	Blyth's Shrike Babbler	70	0	0	0	0	0	20	0	10	0	0	0	0	0	20	80	0	38.67
Pteruthius xanthochlorus	Green Shrike Babbler	60	0	0	0	0	0	20	0	20	0	0	0	0	0	60	40	0	14.3
Alcippe vinipectus	White-browed Fulvetta	80	0	0	0	0	0	10	0	10	0	0	0	0	70	30	0	0	11.95
Alcippe ruficapilla	Spectacled Fulvetta	80	0	0	0	0	0	0	0	20	0	0	0	0	50	50	0	0	13.16
Yuhina diademata	White-collared Yuhina	70	0	0	0	0	0	0	10	20	0	0	0	0	33	33	33	0	12
Yuhina occipitalis	Rufous-vented Yuhina	40	0	0	0	0	0	30	30	0	0	0	0	0	40	40	20	0	13
Conostoma oemodium	Brown-collared Brush-turkey	20	0	0	0	0	0	20	0	30	30	0	0	40	60	0	0	0	83.99
Dicaeum melanoxanthum	Black-fronted Piping-guan	70	0	0	0	0	0	10	10	0	10	0	0	0	0	20	60	20	8.46
Dicaeum ignipectus	Fire-breasted Flowerpecker	20	0	0	0	0	0	40	40	0	0	0	0	0	0	30	70	0	5.72
Aethopyga gouldiae	Mrs Gould's Sunbird	50	0	0	0	0	0	0	50	0	0	0	0	20	30	30	20	0	5
Aethopyga nipalensis	Green-tailed Sunbird	50	0	0	0	0	0	0	50	0	0	0	0	0	30	40	30	0	6.9
Passer rutilans	Russet Sparrow	30	0	0	0	0	0	10	0	60	0	0	0	50	50	0	0	0	18.46
Passer montanus	Eurasian Tree Sparrow	40	0	0	0	0	0	0	0	60	0	0	0	33	33	33	0	0	21.39
Motacilla alba	White Wagtail	100	0	0	0	0	0	0	0	0	0	0	0	100	0	0	0	0	23.93
Motacilla cinerea	Grey Wagtail	100	0	0	0	0	0	0	0	0	0	0	0	100	0	0	0	0	17.15
Anthus hodgsoni	Olive-backed Pipit	100	0	0	0	0	0	0	0	0	0	0	0	100	0	0	0	0	21.3

Anthus sylvanus	Upland Pipit	100	0	0	0	0	0	0	0	0	0	0	0	100	0	0	0	0	31
Prunella strophiata	Rufous-breasted Accentor	60	0	0	0	0	0	20	0	20	0	0	0	100	0	0	0	0	18.08
Carduelis ambigua	Black-headed Greenfinch	0	0	0	0	0	0	0	0	100	0	0	0	50	50	0	0	0	15.38
Uragus sibiricus	Long-tailed Rosefinch	10	0	0	0	0	0	20	0	40	30	0	0	40	30	30	0	0	17.43
Carpodacus nipalensis	Dark-breasted Rosefinch	0	0	0	0	0	0	40	10	40	10	0	0	0	50	50	0	0	22.1
Carpodacus erythrinus	Common Rosefinch	10	0	0	0	0	0	20	10	30	30	0	0	50	50	0	0	0	23.97
Carpodacus pulcherrimus	Himalayan Beautiful Rosefinch	0	0	0	0	0	0	0	0	50	50	0	0	50	50	0	0	0	19.2
Carpodacus thura	Chinese White-browed Rosefinch	0	0	0	0	0	0	30	0	40	30	0	0	100	0	0	0	0	30.99
Pyrrhula nipalensis	Brown Bullfinch	0	0	0	0	0	0	20	10	40	30	0	0	0	40	20	40	0	24.6
Pyrrhula erythaca	Grey-headed Bullfinch	10	0	0	0	0	0	0	10	30	50	0	0	50	50	0	0	0	19
Mycerobas affinis	Collared Grosbeak	10	0	0	0	0	0	20	0	50	20	0	0	33	33	33	0	0	83
Mycerobas melanozanthos	Spot-winged Grosbeak	0	0	0	0	0	0	40	0	40	20	0	0	20	20	40	20	0	62
Mycerobas carnipes	White-winged Grosbeak	10	0	0	0	0	0	60	0	30	0	0	0	0	33	33	33	0	58.84
Emberiza godlewskii	Rufous-headed Chachalaca	30	0	0	0	0	0	0	0	60	10	0	0	100	0	0	0	0	19
Emberiza elegans	Yellow-throated Bunting	30	0	0	0	0	0	0	0	60	10	0	0	0	20	60	20	0	16.84
Ficedula albicilla	Emu	100	0	0	0	0	0	0	0	0	0	0	0	0	30	50	20	0	10.8
Certhia hodgsoni	Hodgson's Treecreeper	60	0	0	0	0	0	0	0	40	0	0	0	40	30	30	0	0	9
Phylloscopus claudiae	Southern Brown Kiwi	80	0	0	0	0	0	20	0	0	0	0	0	0	20	40	40	0	7.63
Phylloscopus ogilviegranti	Black-billed Brush-turkey	100	0	0	0	0	0	0	0	0	0	0	0	0	20	20	60	0	6.4

Table S3 The first two axes and Cumulative Proportion (%) from the principal component analysis of Diet and Forage trait.

Scientific name	Diet-Comp.1	Diet-Comp.2	Forage-Comp.1	Forage-Comp.2
Ithaginis cruentus	-44.34	-3.96	51.49	-2.29
Pucrasia macrolopha	-49.42	-3.90	71.65	13.99
Chrysolophus amherstiae	-20.55	-1.20	71.65	13.99
Picumnus innominatus	42.73	2.57	-39.64	-36.74
Dendrocopos hyperythrus	14.19	0.66	-48.86	33.07
Dendrocopos darjellensis	42.73	2.57	-42.00	-0.36
Picus canus	9.87	-1.60	71.65	13.99
Upupa epops	24.91	0.75	71.65	13.99
Cuculus sparverioides	31.42	-5.72	-48.60	40.90
Cuculus canorus	31.42	-5.72	-21.07	1.33
Psittacula derbiana	-76.21	-11.23	-42.00	-0.36
Columba rupestris	-65.41	43.07	51.49	-2.29
Columba leuconota	-75.81	43.98	51.49	-2.29
Streptopelia orientalis	-71.38	11.72	71.65	13.99
Treron sphenurus	-70.35	-80.31	-24.76	29.25
Accipiter trivirgatus	-29.64	-4.38	-2.66	-19.58
Accipiter virgatus	-29.64	-4.38	11.27	19.61
Lanius tephronotus	24.73	0.81	71.65	13.99
Garrulus glandarius	-25.24	-12.09	27.13	-6.30
Pica pica	-34.93	-5.31	40.67	3.55
Nucifraga caryocatactes	-41.24	34.88	3.29	5.35
Pyrrhocorax pyrrhocorax	-11.25	3.81	35.11	7.96
Corvus corone	-23.62	2.98	62.92	13.70
Corvus macrorhynchos	-37.17	-12.62	71.65	13.99
Pericrocotus solaris	42.73	2.57	-47.57	72.23
Pericrocotus ethologus	21.03	-4.81	-41.17	60.00
Pericrocotus brevirostris	21.94	4.38	-42.00	-0.36

Chelidorhynx hypoxantha	42.73	2.57	-29.31	-20.77
Rhipidura albicollis	42.73	2.57	-41.23	-14.94
Dicrurus macrocercus	-14.84	2.72	-37.52	25.53
Cinclus pallasii	42.73	2.57	3.04	6.98
Monticola solitarius	-19.28	-11.15	47.29	9.97
Myophonus caeruleus	-0.10	-23.20	71.65	13.99
Zoothera dixoni	7.64	-8.48	71.65	13.99
Turdus merula	-13.63	-3.68	27.13	-6.30
Turdus rubrocanus	-13.81	-38.87	23.44	21.62
Turdus mupinensis	-26.29	-33.34	3.29	5.35
Ficedula hodgsonii	-13.81	-38.87	-39.64	-36.74
Ficedula strophiata	42.73	2.57	-29.56	-28.60
Ficedula hyperythra	31.42	-5.72	-33.34	-55.13
Ficedula westermanni	42.73	2.57	-48.60	40.90
Ficedula tricolor	42.73	2.57	-2.66	-19.58
Ficedula sapphira	42.73	2.57	-39.64	-36.74
Eumyias thalassinus	8.81	-22.29	-24.76	29.25
Niltava sundara	20.12	-14.01	21.25	-26.70
Cyornis banyumas	42.73	2.57	-39.64	-36.74
Culicicapa ceylonensis	42.73	2.57	-42.81	6.85
Luscinia calliope	21.94	4.38	21.25	-26.70
Luscinia brunnea	42.73	2.57	41.41	-10.42
Tarsiger cyanurus	18.94	-0.19	21.25	-26.70
Tarsiger chrysaeus	42.73	2.57	21.25	-26.70
Phoenicurus auroreus	-5.10	20.06	21.25	-26.70
Phoenicurus frontalis	42.73	2.57	-42.00	-0.36
Chaimarrornis leucocephalus	18.94	-0.19	51.49	-2.29
Rhyacornis fuliginosa	18.94	-0.19	16.00	-11.37
Hodgsonius phaenicuroides	-2.50	-30.58	71.65	13.99
Saxicola torquatus	10.16	-0.92	71.65	13.99

Saxicola ferreus	30.25	8.10	49.39	3.84
Sitta nagaensis	5.29	19.15	27.13	-6.30
Sitta himalayensis	5.29	19.15	-45.17	5.15
Certhia himalayana	30.25	8.10	-30.89	1.03
Troglodytes troglodytes	1.26	-1.83	21.25	-26.70
Cephalopyrus flammiceps	3.79	1.56	-44.91	12.98
Parus palustris	-15.24	-2.05	-45.94	-18.35
Parus montanus	-4.84	-2.95	-45.94	-18.35
Parus rubidiventris	17.77	13.63	-48.60	40.90
Parus ater	-21.03	13.23	71.65	13.99
Parus dichrous	42.73	2.57	-45.94	-18.35
Parus major	-22.85	-5.15	-45.43	-2.68
Parus monticolus	-15.24	-2.05	-42.81	6.85
Sylviparus modestus	17.77	13.63	-45.17	5.15
Aegithalos iouschistos	11.55	5.28	-38.36	2.42
Hirundo rupestris	42.73	2.57	6.97	-22.58
Delichon nipalense	42.73	2.57	-21.97	23.30
Pycnonotus xanthorrhous	-50.08	-36.10	21.25	-26.70
Hypsipetes leucocephalus	-41.44	-31.59	-46.24	42.61
Zosterops palpebrosus	-22.23	-8.08	-48.34	48.73
Phylloscopus fuscatus	17.77	13.63	-39.64	-36.74
Phylloscopus affinis	42.73	2.57	21.25	-26.70
Phylloscopus subaffinis	42.73	2.57	-10.73	17.30
Phylloscopus armandii	42.73	2.57	-2.66	-19.58
Phylloscopus pulcher	24.58	-0.25	-48.60	40.90
Phylloscopus proregulus	42.73	2.57	-38.36	2.42
Phylloscopus inornatus	30.25	8.10	-30.89	1.03
Phylloscopus borealis	42.73	2.57	-38.36	2.42
Phylloscopus trochiloides	18.94	-0.19	-42.00	-0.36
Phylloscopus magnirostris	42.73	2.57	-48.08	56.57

Seicercus burkii	42.73	2.57	-50.14	-6.09
Garrulax cineraceus	7.64	-8.48	71.65	13.99
Garrulax bieti	-13.81	-38.87	71.65	13.99
Garrulax maximus	21.94	4.38	49.39	3.84
Garrulax elliotii	21.94	4.38	21.25	-26.70
Garrulax affinis	-36.42	-55.45	6.97	-22.58
Liocichla phoenicea	-28.63	-5.71	21.25	-26.70
Pomatorhinus erythrocnemis	42.73	2.57	71.65	13.99
Pomatorhinus ruficollis	18.94	-0.19	-33.34	-55.13
Chrysomma poecilotis	42.73	2.57	-39.64	-36.74
Pteruthius flaviscapis	7.64	-8.48	-48.08	56.57
Pteruthius xanthochlorus	-4.84	-2.95	-49.11	25.24
Alcippe vinipectus	18.94	-0.19	-35.44	-49.00
Alcippe ruficapilla	17.77	13.63	-39.64	-36.74
Yuhina diademata	8.70	12.22	-42.00	-0.36
Yuhina occipitalis	-18.42	-26.52	-41.23	-14.94
Conostoma oemodium	-48.51	5.29	11.17	-34.84
Dicaeum melanoxanthum	11.96	-6.22	-41.68	44.33
Dicaeum ignipectus	-38.80	-36.22	-48.34	48.73
Aethopyga gouldiae	-2.64	-4.48	-18.97	-4.80
Aethopyga nipalensis	-2.64	-4.48	-43.07	-0.98
Passer rutilans	-43.45	27.45	21.25	-26.70
Passer montanus	-32.15	35.74	-2.66	-19.58
Motacilla alba	42.73	2.57	71.65	13.99
Motacilla cinerea	42.73	2.57	71.65	13.99
Anthus hodgsoni	42.73	2.57	71.65	13.99
Anthus sylvanus	42.73	2.57	71.65	13.99
Prunella strophiata	-4.84	-2.95	71.65	13.99
Carduelis ambigua	-82.06	57.85	21.25	-26.70
Uragus sibiricus	-60.99	10.82	4.87	-16.45

Carpodacus nipalensis	-71.89	-8.98	-39.64	-36.74
Carpodacus erythrinus	-57.58	3.88	21.25	-26.70
Carpodacus pulcherrimus	-71.64	34.73	21.25	-26.70
Carpodacus thura	-72.29	2.53	71.65	13.99
Pyrrhula nipalensis	-70.06	9.41	-40.71	0.72
Pyrrhula erythaca	-55.76	22.26	21.25	-26.70
Mycerobas affinis	-63.07	15.44	-2.66	-19.58
Mycerobas melanozanthos	-73.21	-6.66	-21.07	1.33
Mycerobas carnipes	-62.55	-30.57	-42.00	-0.36
Emberiza godlewskii	-42.54	36.64	71.65	13.99
Emberiza elegans	-42.54	36.64	-45.43	-2.68
Ficedula albicilla	42.73	2.57	-43.33	-8.81
Certhia hodgsoni	-7.19	24.68	4.87	-16.45
Phylloscopus claudiae	20.12	-14.01	-44.91	12.98
Phylloscopus ogilviegranti	42.73	2.57	-44.40	28.64
**Cumulative Proportion (%)**	**0.67**	**0.83**	**0.65**	**0.85**

Table S4 Relationships between elevational distance and observed multiple dimensional β-diversity (i.e., taxonomic, phylogenetic, and functional β-diversity) based on liner regression models.

Component	S	R^2	p
βsim	3.15E-04	0.69	<0.001
βsne	-1.89E-05	-0.02	0.23
βsor	2.96E-04	0.87	<0.001
βphylosim	2.20E-04	0.59	<0.001
βphylosne	7.23E-06	-0.03	0.3
βphylosor	2.32E-04	0.71	<0.001
βfuncsim	3.99E-05	0.04	0.03
βfuncsne	-1.39E-05	-0.03	0.109
βfuncsor	2.60E-05	-0.002	0.38

Table S5 Relationships between elevational distance and standardized multiple dimensional β-diversity (i.e., taxonomic, phylogenetic, and functional β-diversity) based on liner regression models.

	**S**	**R^2**	**p**
ses.βsim	4.97E-03	0.69	<0.001
ses.βsne	-7.45E-04	-0.03	0.29
ses.βsor	5.25E-03	0.87	<0.001
ses.β phylosim	3.66E-03	0.58	<0.001
ses.β phylosne	1.24E-04	-0.04	0.612
ses.β phylosor	4.15E-03	0.69	<0.001
ses.βfuncsim	8.27E-04	0.02	0.09
ses.βfuncsne	-2.60E-04	-0.03	0.15
ses.βfuncsor	6.10E-04	0.03	0.01

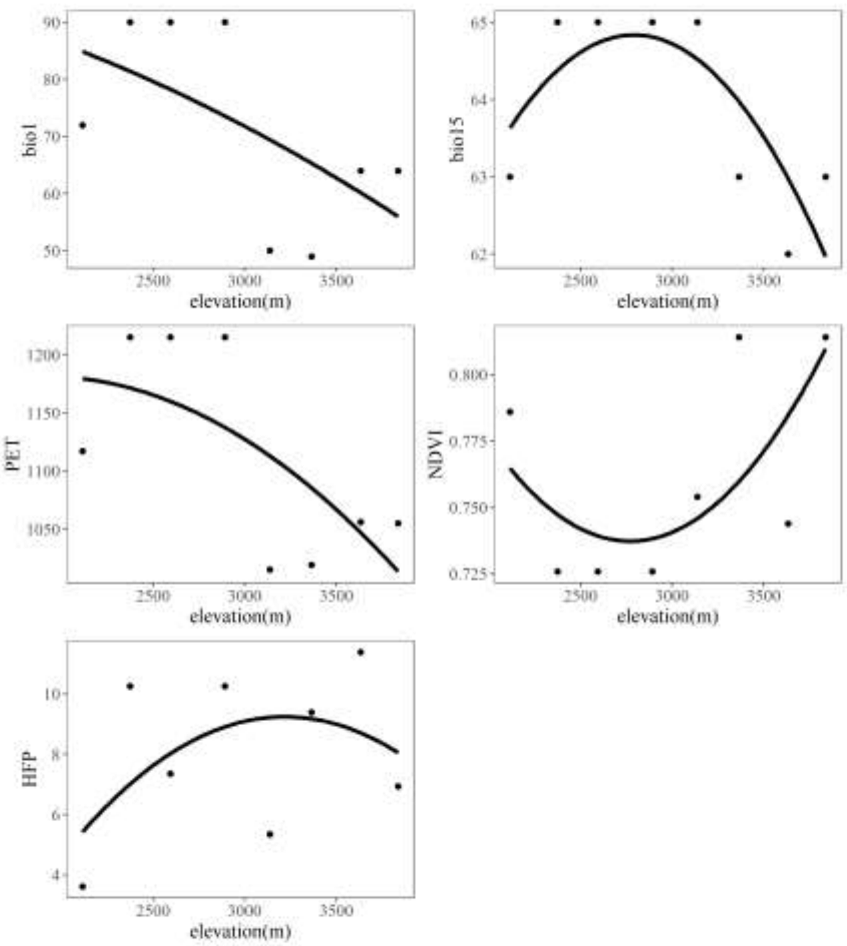

Figure S1 Relationships between elevational and environmental variables. bio1: annual mean temperature; bio15: precipitation seasonality; HFP: human footprints; NDVI: normalized difference vegetation index; PET: potential evapotranspiration.
